# Eosinophil-associated intestinal immune responses following SARS-CoV-2 infection in *K18-hACE2* mice

**DOI:** 10.1371/journal.pone.0355607

**Published:** 2026-08-12

**Authors:** Shun Tamaki, Rei Kawashima, Takayuki Uematsu, Fumitaka Kawakami, Motoki Imai, Yoshifumi Kurosaki, Toshihide Matsumoto, Shotaro Maehana, Yusuke Hara, Setsuko Sugawara, Hiroki Izawa, Takafumi Ichikawa, Hidero Kitasato, Hideaki Hanaki, Makoto Kubo

**Affiliations:** 1 Department of Biochemistry, Kitasato University School of Allied Health Sciences, Sagamihara, Kanagawa, Japan; 2 Department of Regulation Biochemistry, Kitasato University Graduate School of Medical Sciences, Sagamihara, Kanagawa, Japan; 3 Regenerative Medicine and Cell Design Research Facility, Kitasato University School of Allied Health Sciences, Sagamihara, Kanagawa, Japan; 4 Laboratory of Infection Immunology, Department of Infection Control and Immunology, O̅mura Satoshi Memorial Institute, Kitasato University, Minato-ku, Tokyo, Japan; 5 Biomedical Laboratory, Division of Biomedical Research, Kitasato University Medical Center, Kitamoto-shi, Saitama, Japan; 6 Department of Health Science, Kitasato University School of Allied Health Sciences, Sagamihara, Kanagawa, Japan; 7 Department of Molecular Diagnostics, Kitasato University School of Allied Health Sciences, Sagamihara, Kanagawa, Japan; 8 Department of Applied Tumor Pathology, Kitasato University Graduate School of Medical Sciences, Sagamihara, Kanagawa, Japan; 9 Department of Clinical Chemistry, Kitasato University School of Allied Health Sciences, Sagamihara, Kanagawa, Japan; 10 Department of Pathology, School of Allied Health Sciences, Kitasato University, Sagamihara, Kanagawa, Japan; 11 Department of Microbiology, School of Allied Health Sciences, Kitasato University, Sagamihara, Kanagawa, Japan; 12 Department of Environmental Microbiology, Kitasato University Graduate School of Medical Sciences, Sagamihara, Kanagawa, Japan; 13 Department of Gastroenterology, Kitasato University Graduate School of Medical Sciences, Sagamihara, Kanagawa, Japan; 14 Department of Gastroenterology, Kitasato University School of Medicine, Sagamihara, Kanagawa, Japan; 15 Infection Control Research Center, Ōmura Satoshi Memorial Institute, Kitasato University, Minato-ku, Tokyo, Japan; São Paulo State University Institute of Biosciences Languages and Exact Sciences: Universidade Estadual Paulista Julio de Mesquita Filho Instituto de Biociencias Letras e Ciencias Exatas, BRAZIL

## Abstract

**Background:**

Coronavirus disease 2019 (COVID-19), caused by severe acute respiratory syndrome coronavirus 2 (SARS-CoV-2), is frequently associated with gastrointestinal manifestations; however, intestinal immune responses following SARS-CoV-2 infection remain incompletely understood. This study aimed to characterize eosinophil-associated intestinal immune responses in SARS-CoV-2-infected *K18-hACE2* transgenic mice.

**Methods:**

Small intestinal tissues from infected mice were analyzed using cytokine and chemokine PCR arrays, qRT-PCR, immunohistochemistry, and histological analyses.

**Results:**

SARS-CoV-2 infection was associated with marked upregulation of *Il5* mRNA in small intestinal tissues, along with increased expression of eosinophil-related cytokines and chemokines, including *Ccl24* and *Ccl11*. Immunohistochemical analyses showed eosinophil accumulation in the lamina propria, accompanied by increased signals for eosinophil-associated granule proteins, including major basic protein and eosinophil peroxidase. Additional qRT-PCR analyses showed increased expression of *Prg2* and *Epx*, further supporting eosinophil granule protein-associated responses. Charcot-Leyden crystals and increased *Galectin-10* expression were also observed. Temporal analyses showed an acute *Il5*/*Ccl24*-related response followed by delayed *Ccl11* upregulation during later stages.

**Conclusions:**

These findings indicate that SARS-CoV-2 infection is associated with eosinophil-associated intestinal immune responses in *K18-hACE2* mice. Although eosinophil activation or intestinal allergy was not directly demonstrated, this study supports virus-associated eosinophil-related intestinal responses as a possible framework for understanding allergy-like intestinal immune alterations during SARS-CoV-2 infection.

## Introduction

Coronavirus disease 2019 (COVID-19) is caused by severe acute respiratory syndrome coronavirus 2 (SARS-CoV-2). COVID-19 is now recognized as a systemic disease affecting multiple organ systems beyond the respiratory tract [1]. In addition to pulmonary involvement, patients frequently present with extrapulmonary manifestations, including cardiovascular, renal, and gastrointestinal (GI) disorders. Among these, GI symptoms such as diarrhea, nausea, vomiting, and abdominal pain have been reported in a substantial proportion of cases [1–3]. Notably, these clinical features often appear during the early stages of infection and may precede respiratory symptoms. The presence of GI manifestations has been associated with increased disease severity and the development of long-term sequelae, commonly referred to as long COVID. Together, these observations indicate that the GI tract plays an important role in the heterogeneous clinical presentation of COVID-19 [1,4,5].

The intestine represents a potential primary site of SARS-CoV-2 infection, as angiotensin-converting enzyme 2 (ACE2), the main viral entry receptor, is abundantly expressed in intestinal epithelial cells [6,7]. This molecular distribution provides a strong biological basis for direct viral infection of the GI mucosa [8]. Consistent with this concept, experimental studies using human intestinal organoids and transgenic mouse models have demonstrated productive viral replication in intestinal epithelial cells [9,10]. Furthermore, viral RNA and proteins have been detected in intestinal tissues and fecal samples from patients with COVID-19, suggesting sustained intestinal involvement even after clearance of respiratory infection [1,11]. Despite this evidence, the immunological and pathological consequences of intestinal SARS-CoV-2 infection remain poorly understood. In particular, the contribution of local mucosal immune responses to both acute GI symptoms and persistent manifestations observed in long COVID has not been fully elucidated [2,5].

To address this knowledge gap, the present study characterized immune and inflammatory changes in the small intestinal tissues of *K18-hACE2* transgenic mice following SARS-CoV-2 infection. We focused on alterations in cytokine and chemokine expression profiles, histological features of mucosal inflammation, and immune pathways potentially associated with type 2 inflammatory responses. Through this approach, we aimed to provide insight into intestinal immune processes that may underlie gastrointestinal involvement in COVID-19.

## Materials and methods

### Mice

This study used heterozygous *K18-hACE2* transgenic mice expressing human ACE2. Human ACE2 is expressed under the regulation of the cytokeratin-18 promoter. The *K18* promoter predominantly directs gene expression to multiple epithelium-lined tissues and neurons [12–15]. The mice used in these previous studies were congenic on the C57BL/6 background (*034860-B6.Cg-Tg(K18-ACE2)2Prlman/J*) and were obtained from Jackson Laboratory (Bar Harbor, ME). During the infection period, physiological parameters including body weight, fecal condition, and general behavior were monitored twice daily. Humane endpoints were predefined before the start of the study. Animals were scheduled for euthanasia if they exhibited unexpected severe distress, defined as body weight loss exceeding 20% within 2–3 days or 25% within 5 days. However, no animals met these criteria during the course of the study. All procedures were conducted in accordance with institutional guidelines for animal care and use. No anesthesia was used. Euthanasia was performed by cervical dislocation by an animal experimenter with more than 10 years of experience.

All animal experiments were conducted in accordance with the approved protocols and institutional guidelines of Kitasato University, and all efforts were made to minimize animal suffering. The animal study protocol was approved by the Institutional Animal Care and Use Committee of Kitasato University (protocol codes: 2020−8, 2021−9, and 2022−9) and by the Genetic Modification Experiment Safety Committee of Kitasato University (approval numbers: 4557 and 5205). This study did not involve human participants; therefore, informed consent was not applicable.

### Virus

The SARS-CoV-2 strain *hCoV-19/Japan/TY/WK-521* (WK-521, Pango Lineage A) [16], which was isolated from patients with COVID-19, was provided by the National Institute of Infectious Diseases (Tokyo, Japan) and passaged in VeroE6/TMPRSS2 cells (ATCC CRL-1586). The VeroE6/TMPRSS2 cells were maintained in Dulbecco’s Modified Eagle Medium (Fujifilm Wako Pure Chemical, Osaka, Japan) supplemented with 10% fetal bovine serum (Life Technologies, Carlsbad, CA, the USA).

### Viral infection in mice

Transgenic *K18-hACE2* heterozygous mice, aged 8–12 weeks, were anesthetized using a combination of medetomidine hydrochloride, midazolam, and butorphanol tartrate. They subsequently received intranasal administration of 5 × 10^4^ plaque-forming units (PFUs) (high titer) or 1 × 10^3^ PFUs (low titer) of SARS-CoV-2 (strain *hCoV-19/Japan/TY/WK-521*). In the control group, uninfected mice were administered with Dulbecco’s Modified Eagle Medium and antibiotics (100 IU/mL of penicillin and 100 µg/mL of streptomycin, Fujifilm Wako Pure Chemical). At 7 days post-infection in the high-titer group or at 2, 4, 7, 14, 21, and 28 days post-infection in the low-titer group, the mice were humanely euthanized. Then, intestinal tissue samples were collected (S1 File). All experiments were conducted in a secure animal biosafety level 3 facility.

### Quantitative reverse-transcriptase polymerase chain reaction (qRT-PCR), RT^2^ profiler PCR array, and agarose gel electrophoresis

Total RNA was extracted from tissue samples using TRIzol Reagent (Thermo Fisher Scientific, Waltham, MA, USA) according to the manufacturer’s instructions. RNA concentration and purity were assessed spectrophotometrically (NanoDrop, Thermo Fisher Scientific), and samples with an A260/A280 ratio between 1.8 and 2.0 were used for subsequent analyses. Complementary DNA (cDNA) was synthesized from 1 μg of total RNA using the PrimeScript RT Reagent Kit (Takara Bio Inc., Shiga, Japan) following the manufacturer’s protocol. Quantitative real-time PCR (qRT-PCR) was performed using SYBR Select Master Mix (Thermo Fisher Scientific) in a total reaction volume of 20 μL containing 10 μL SYBR Master Mix, 0.4 μM of each primer, and 2 μL of cDNA template. Amplification was carried out using the ABI 7500 Real-Time PCR System (Applied Biosystems, Foster City, CA, USA). The thermal cycling conditions were as follows: initial denaturation at 95°C for 10 min, followed by 40 cycles of denaturation at 95°C for 15 s and annealing/extension at 60°C for 1 min. A melting curve analysis was performed to confirm amplification specificity. All reactions were performed in technical triplicate. The gene-specific primer sequences were as follows:

*Ccl11* (forward: 5′-tccacagcgcttctattcct; reverse: 5′-ctatggctttcagggtgcat),
*Ccl24* (forward: 5′-ctgtgaccatcccctcatct; reverse: 5′-tcttatggcccttcttggtg),
*Ccr3* (forward: 5′-ccactgtactccctggtgttca; reverse: 5′-ggacagtgaagagaaagagcagg),
*Epx* (forward: 5′-ctgtctcctgactaaccgctct; reverse: 5′-tcagcggctaggcgattgtgtt),
*Il5* (forward: 5′-gatgaggcttcctgtccctact; reverse: 5′-tgacaggttttggaatagcatttcc),
*Il5ra* (forward: 5′-gacaccaaaggtgacctcacag; reverse: 5′-ctgtctctgtggtcattgcggt),
*Il4* (forward: 5′-atcatcggcattttgaacgaggtc; reverse: 5′-accttggaagccctacagacga),
*Il13* (forward: 5′-aacggcagcatggtatggagtg; reverse: 5′-tgggtcctgtagatggcattgc),
*Il25* (forward: 5′-cattcttggcaatgatcgtg; reverse: 5′-ctccacttcagccactcctc),
*Il33* (forward: 5′-tggcctcaccataagaaagg; reverse: 5′-catgcttggtacccgatttt),
*Prg2* (forward: 5′-caagacctgtcgctacctccta; reverse: 5′-gcggactggattccgaagttaac),
*Tslp* (forward: 5′-cggatggggctaacttacaa; reverse: 5′-tcctcgatttgctcgaactt),
*Galectin10* (forward: 5′-gtggatttccacaccgagat; reverse: 5′-ctgttcatgaccacccaatg),
*Gapdh* (forward: 5′-catcactgccacccagaagact; reverse: 5′-atgccatgtagcttcccgttca).

Relative gene expression levels were calculated using the 2^−ΔΔCT^ method, with normalization to the housekeeping gene Gapdh. The RT^2^ Profiler PCR Array was used as an exploratory screening analysis to identify candidate immune-related molecules. Selected molecules were subsequently examined by qRT-PCR using independent biological samples. For the RT^2^ Profiler PCR Array (QIAGEN N.V., Venlo, The Netherlands), cDNA samples were mixed with SYBR Select Master Mix according to the manufacturer’s protocol and distributed into each well of the array plate. Amplification was performed using the ABI 7500 Real-Time PCR System under the recommended cycling conditions, and data were analyzed using the manufacturer-provided Excel-based analysis template. The list of target genes included in the RT^2^ Profiler PCR Array is provided in S2 File. To confirm amplification specificity, selected PCR products obtained from the RT^2^ Profiler PCR Array were separated by 2% agarose gel electrophoresis in Tris-acetate-EDTA (TAE) buffer. DNA bands were stained with 0.5 μg/mL ethidium bromide and visualized using a gel documentation system (Bio-Rad Laboratories, Hercules, CA, USA). For the RT^2^ Profiler PCR Array, small intestinal samples from two of the six mice in each group of the high-titer infection experiment (5 × 10⁴ PFU/mouse; D0 and D7) were used as an exploratory screening analysis. Subsequent qRT-PCR analyses were performed using six biological replicates per group.

### Histological analysis

The tissues were immediately fixed for 24 h in 10% Neutral Buffered Formalin (Fujifilm Corporation, Tokyo, Japan), processed, and paraffin-embedded. Three-micrometer sections were washed in xylene and rehydrated in graded ethanol solutions. For antigen retrieval, paraffin-embedded sections were incubated in 10 mmol/L citric buffer at 95°C for 15 min. Endogenous peroxidase activity was blocked with 3% H_2_O_2_ in phosphate-buffered saline (PBS), followed by permeabilization with 0.5% Triton X-100 in PBS. Sections were incubated overnight at 4°C for 16 h with the following primary antibodies diluted in DAKO Antibody Diluent with Background Reducing Components (Agilent Technologies, Santa Clara, CA, USA): a rabbit polyclonal antibody against eosinophil granule major basic protein (MBP; MPRG2) (Biorbyt Ltd., Cambridge, UK; 1:200), a rabbit anti-SARS-CoV-2 spike antibody (40589-T62; Sino Biological, Inc., Beijing, China; 1:500), and a rabbit anti-EPX antibody (31743T; Cell Signaling Technology, Danvers, MA, USA; 1:400). After washing, sections were incubated with DAKO EnVision+ System-HRP Labeled Polymer Anti-Rabbit (Agilent Technologies) for 30 min at 25°C. Immunoreactivity was visualized using the ImmPACT DAB Substrate Kit (Vector Laboratories, Inc., Newark, CA, USA). For negative controls, sections were processed in parallel with omission of the primary antibody and incubated with antibody diluent alone to confirm the specificity of staining. An optical microscope (Olympus Corporation, Tokyo, Japan) was used to assess and photograph the tissues. For quantitative histological analysis, well-oriented villi were selected from hematoxylin and eosin–stained sections, and villus height was measured from the villus tip to the crypt–villus junction using ImageJ software (National Institutes of Health, Bethesda, MD, USA). Multiple villi were measured from each animal, and the mean value for each animal was used as one biological replicate for statistical analysis. For immunohistochemical quantification, MBP- and EPX-positive cells were counted in the lamina propria of well-oriented villi. Positive cells were defined as cells showing clear DAB-positive staining with appropriate cellular morphology. Multiple villi were evaluated from each animal under the same microscopic conditions, and the mean value for each animal was used as one biological replicate. Quantification was performed using the same criteria and imaging conditions across all groups. Histological and immunohistochemical analyses were performed using small intestinal samples from six biological replicates per group.

### Papanicolaou and hematoxylin–eosin (H&E) staining

The tissues were immediately fixed for 24 h in 10% Neutral Buffered Solution (Fujifilm Corporation), processed, and paraffin-embedded. Three-micrometer sections were washed in xylene and rehydrated in gradient ethanol aliquots. Papanicolaou staining was performed using the Papanicolaou Stain Kit (Scy Tek Laboratories Inc., Logan, UT, the USA). For hematoxylin–eosin (H&E) staining, sections were stained with hematoxylin, rinsed in running tap water, differentiated when necessary, and counterstained with eosin. After dehydration through graded ethanol and clearing in xylene, the sections were mounted with a permanent mounting medium.

### Statistical analysis

Quantitative data are expressed as mean ± standard deviation (SD). Statistical analyses were performed using GraphPad Prism version 10.0 (GraphPad Software Inc., San Diego, CA, USA). Because the sample sizes were relatively small and normal distribution could not be assumed, non-parametric tests were used for the statistical analyses. For the high-titer infection experiment (5 × 10⁴ PFU/mouse), comparisons between two independent groups (D0 and D7) were performed using the Mann–Whitney U test. For the low-titer time-course experiment (1 × 10^3^ PFU/mouse; D0, D2, D4, D7, D14, D21, and D28; independent cohorts), comparisons among multiple independent groups were performed using the Kruskal–Wallis test followed by Dunn’s multiple comparisons test. A p-value < 0.05 was considered statistically significant. This study was not preregistered; however, all experimental procedures and statistical analyses were determined prior to data collection.

## Results

### Comprehensive analysis of the expression of cellular response mediator genes

**Fig 1:**
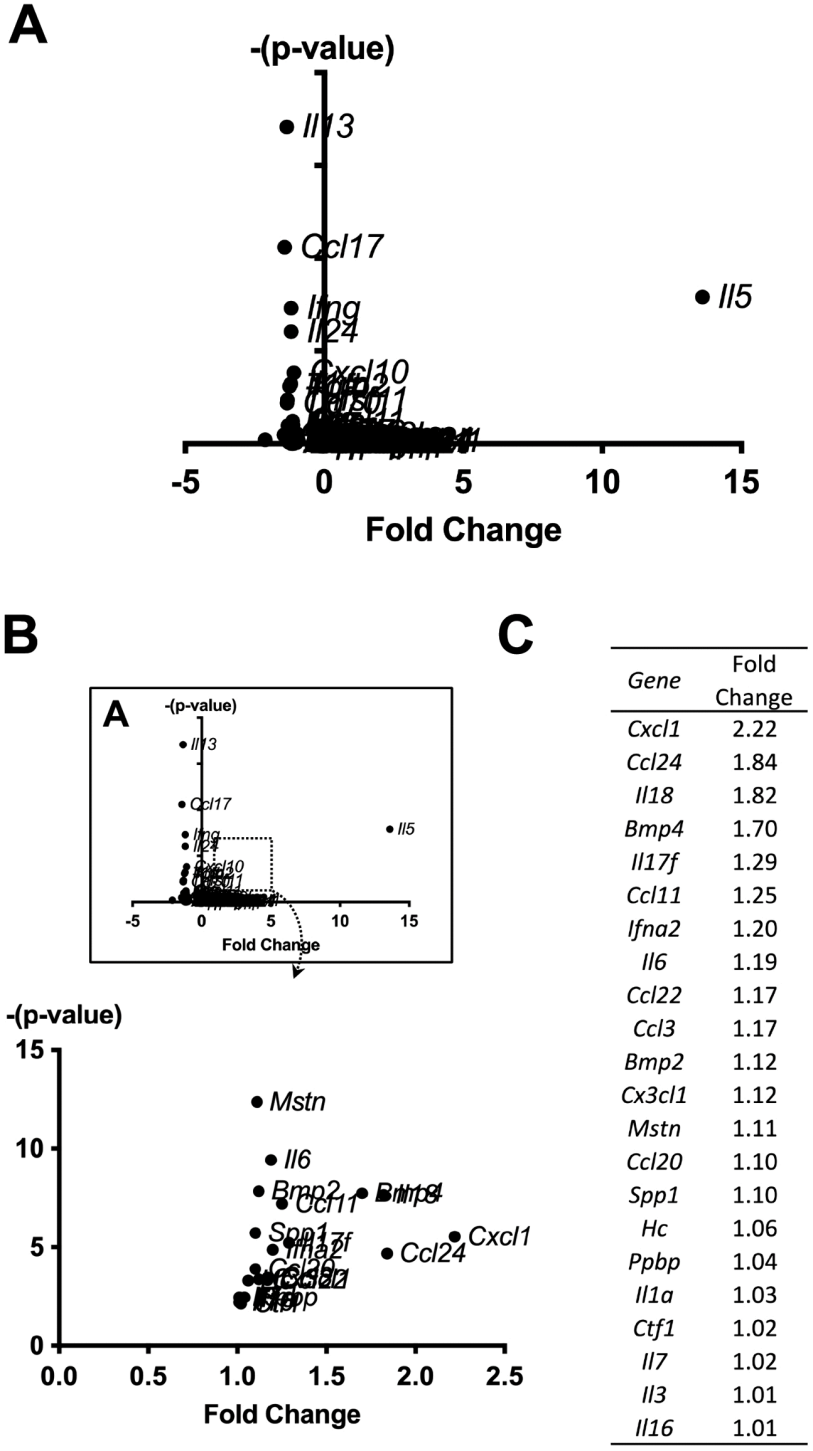
RT^2^ profiler PCR array of small intestinal cytokines and chemokines after SARS-CoV-2 infection. (A) Quantitative reverse-transcriptase polymerase chain reaction (qRT-PCR) analysis of the 84 cytokine and chemokine genes in the small intestine in the uninfected state and at 7 days after infection with SARS-CoV-2 (5 × 10^4^ PFU/mouse) in the *K18-hACE2*-Tg mice. *GAPDH* was used as a loading control. n = 2 biological replicates. (B) Part of the RT^2^ array was enlarged (dotted line of Fig 1A). (C) Multiple genes with a high expression next to *Il5* were listed.

To examine the effects of SARS-CoV-2 infection (5 × 10^4^ PFU/mouse) on the GI tract in *K18-hACE2*-Tg mice, the mRNA expression of 84 cytokine and chemokine genes in the small intestinal tissues on day 7 of infection was comprehensively analyzed using the RT^2^ Profiler PCR Array (S2 File). Results showed that the *Il5* mRNA expression was approximately 13-fold higher in the infected group (day 7) than in the uninfected group (day 0) (Fig 1A). Visualization of the *Il5* PCR product obtained using the RT^2^ Profiler PCR Array via agarose gel electrophoresis confirmed an increase in the band area in the infected group (S1 Fig). Further, the RT^2^ Profiler PCR Array revealed that *Il5* was not the only gene whose expression was elevated in the infected group. However, the in-crease in the expression was low. Fig 1B shows an enlarged view of the gene clusters showing an increased expression. If the highly expressed genes were ranked in de-scending order of expression, *Cxcl1* (2.22-fold), *Ccl24* (1.84-fold), *Il18* (1.82-fold), *Bmp4* (1.70-fold), *Il17f* (1.29-fold), and *Ccl11* (1.25-fold) were upregulated after *Il5* (Fig 1C). Based on these findings, SARS-CoV-2 infection may induce inflammation-related factors, including *Il5*, in the GI mucosa.

### Eosinophil-associated cytokine and chemokine responses

**Fig 2:**
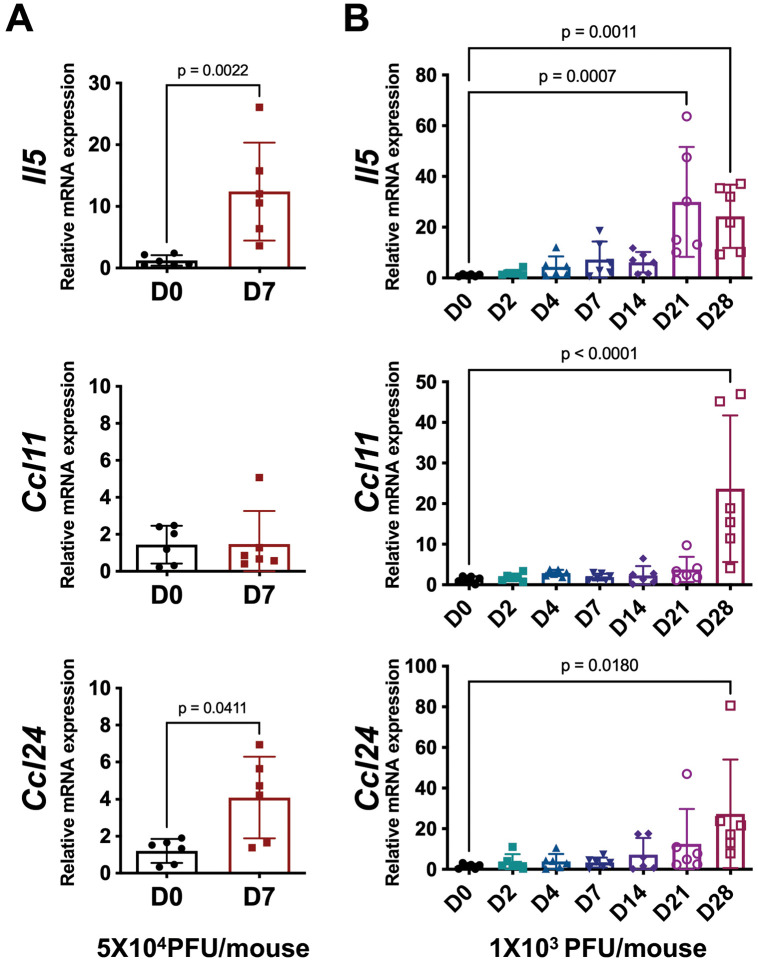
Gene expression analysis of eosinophil-associated factors in the small intestinal tissues after SARS-CoV-2 infection. Quantitative reverse-transcriptase polymerase chain reaction (qRT-PCR) analysis of *Il5*, *Ccl11* (eotaxin), and *Ccl24* (eotaxin-2) in the small intestine in the uninfected state (D0), at 7 days (D7) after SARS-CoV-2 infection (5 × 10^4^ PFU/mouse) (A), and at 0, 2, 4, 7, 14, 21, and 28 days (D2, D4, D7, D14, D21, and D28) after infection with SARS-CoV-2 (1 × 10^3^ PFU/mouse) (B) in the *K18-hACE2*-Tg mice. *Gapdh* was used as a loading control. n = 6 biological replicates.

Fig 1C shows that several molecules with increased expression were related to eosinophil infiltration. Therefore, real-time PCR was used to compare the mRNA expression of these molecules in the small intestinal tissues of *K18-hACE2*-Tg mice infected with SARS-CoV-2 at a high titer (5 × 10⁴ PFU/mouse) or low titer (1 × 10^3^ PFU/mouse). The expression of *Il5* and *Ccl24* was upregulated in the high-titer infected group on day 7 (Fig 2A). In contrast, in the low-titer infected group, their expression was not elevated on day 7 but increased on days 21 and 28 (Fig 2B). Interestingly, *Ccl11* expression remained unchanged in the high-titer infected group (Fig 2A), whereas it was upregulated in the low-titer infected group on day 28. By approximately day 7 after infection, SARS-CoV-2 spike protein was detected in the lung tissues of the low-titer infected group (S2 Fig); however, this finding does not indicate the presence of infectious viral particles. In contrast, SARS-CoV-2 spike protein was not detected in intestinal tissues at the early stage of infection (S2 Fig). These results suggest that eosinophil-associated inflammatory responses in the intestinal mucosa may occur during SARS-CoV-2 infection, even in the absence of detectable viral antigen in the intestinal tissue.

### Eosinophil infiltration into the small intestinal mucosa

**Fig 3:**
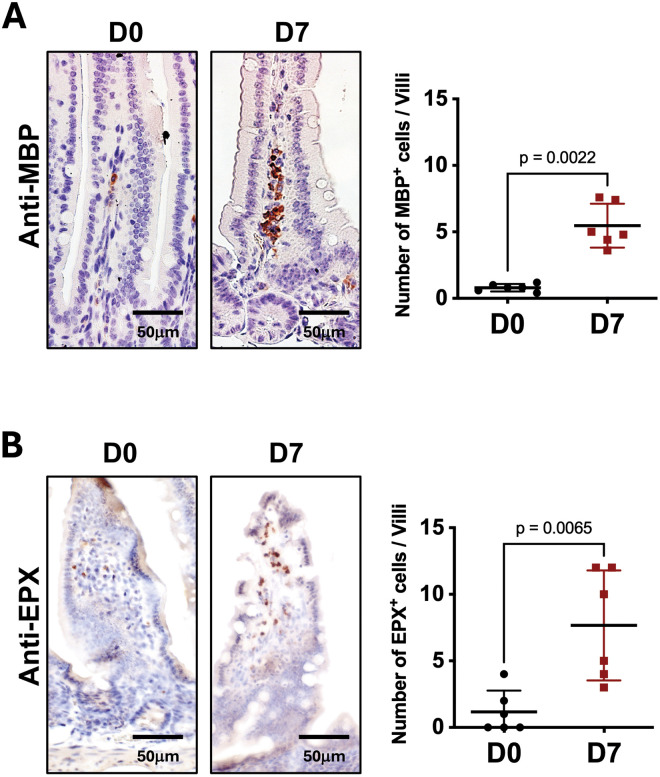
Detection of eosinophil marker-positive cells in the small intestinal mucosa after SARS-CoV-2 infection. (A) Immunohistochemistry for major basic protein (MBP) in the small intestine of uninfected mice (D0) and mice at 7 days after SARS-CoV-2 infection (D7; 5 × 10⁴ PFU/mouse) in *K18-hACE2-Tg* mice. The graph shows the quantification of MBP-positive cells per villus. (B) Immunohistochemistry for eosinophil peroxidase (EPX) in the small intestine of uninfected mice (D0) and mice at 7 days after SARS-CoV-2 infection (D7; 5 × 10⁴ PFU/mouse) in *K18-hACE2*-Tg mice. The graph shows the quantification of EPX-positive cells per villus. n = 6 biological replicates per group. Scale bars = 50 μm.

As depicted in Fig 2, SARS-CoV-2 infection might induce eosinophil-associated responses in the small intestine. Therefore, the presence of eosinophils in the small intestine of SARS-CoV-2-infected *K18-hACE2*-Tg mice was evaluated. Immunohistochemical staining of small intestinal tissues using antibodies against the eosinophil markers major basic protein (MBP) and eosinophil peroxidase (EPX) revealed positive signals in the lamina propria region. Quantitative analysis showed that the number of MBP-positive cells was higher in the infected group on day 7 than in the uninfected group on day 0 (Fig 3A). Similarly, the number of EPX-positive cells was higher in the infected group on day 7 than in the uninfected group on day 0 (Fig 3B). These findings suggest that SARS-CoV-2 infection promotes eosinophil infiltration into the small intestinal mucosa.

### Additional analysis of eosinophil-associated gene expression

**Fig 4:**
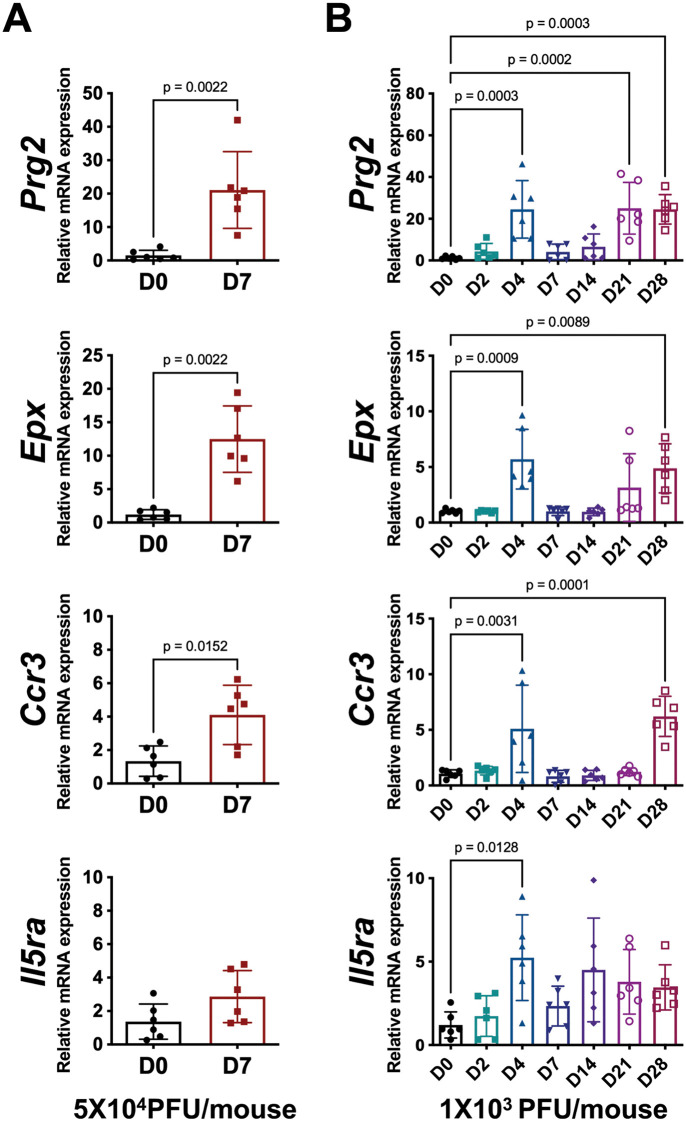
Expression patterns of eosinophil-associated genes in the small intestinal mucosa after SARS-CoV-2 infection. Quantitative reverse-transcriptase polymerase chain reaction (qRT-PCR) analysis was performed to evaluate the mRNA expression of *Prg2*, *Epx*, *Ccr3*, and *Il5ra* in the small intestine of *K18-hACE2-Tg* mice after SARS-CoV-2 infection. *Prg2* and *Epx* were examined as eosinophil granule protein-associated genes, whereas *Ccr3* and *Il5ra* were examined as genes related to eosinophil recruitment and IL-5 responsiveness, respectively. In the high-titer infection experiment, uninfected mice (D0) and mice at 7 days after infection (D7; 5 × 10⁴ PFU/mouse) were analyzed. In the low-titer infection experiment, mice infected with 1 × 10^3^ PFU/mouse were analyzed over the indicated time course. *Gapdh* was used as an internal control. Data are presented as mean ± SD. n = 6 biological replicates per group.

Based on the eosinophil-associated cytokine and chemokine responses shown in Fig 2, we further examined the mRNA expression of eosinophil-related genes, including Prg2, Epx, Ccr3, and Il5ra, in the small intestinal tissues of *K18-hACE2-Tg* mice infected with SARS-CoV-2. In the high-titer infected group, *Prg2* and *Epx*, which encode eosinophil granule-associated proteins, were increased on day 7 after infection (Fig 4). In the low-titer infected group, *Prg2* and *Epx* showed a biphasic increase, with elevated expression observed around days 4 and 28 after infection (Fig 4). *Ccr3*, a chemokine receptor associated with eosinophil recruitment, also showed a similar increase after infection, particularly in the low-titer infected group (Fig 4). In contrast, *Il5ra*, which is involved in IL-5 responsiveness, showed only a modest increasing trend and did not reach statistical significance (Fig 4). These findings further support the presence of eosinophil-associated intestinal responses following SARS-CoV-2 infection.

### Eosinophil-associated cytokine responses

**Fig 5:**
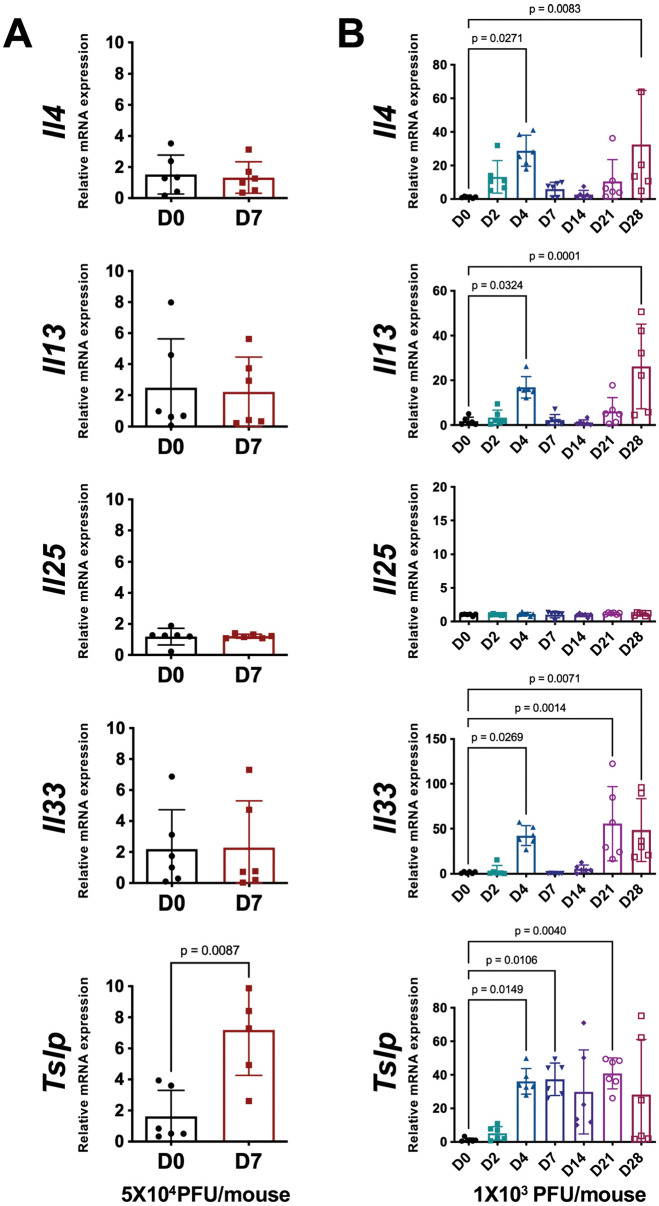
Gene expression analysis of type 2 cytokines and epithelial alarmins in the small intestinal tissues after SARS-CoV-2 infection. Quantitative reverse-transcriptase polymerase chain reaction (qRT-PCR) analysis of *Il4*, *Il13*, *Il25*, *Il33*, and *Tslp* mRNA expression in the small intestine of uninfected mice (D0), mice at 7 days after SARS-CoV-2 infection (D7; 5 × 10⁴ PFU/mouse) (A), and mice at 0, 2, 4, 7, 14, 21, and 28 days after SARS-CoV-2 infection (D0, D2, D4, D7, D14, D21, and D28; 1 × 10^3^ PFU/mouse) (B) in *K18-hACE2*-Tg mice. *Gapdh* was used as an internal control. Data are shown as individual values with mean ± SD. n = 6 biological replicates per group.

Figs 2−4 show that SARS-CoV-2 infection may be associated with eosinophil accumulation and eosinophil-associated gene expression in the small intestine. To examine cytokine responses related to eosinophil-associated intestinal inflammation, real-time PCR was conducted to compare the mRNA expression of type 2 cytokines and epithelial alarmins in the small intestinal tissues of *K18-hACE2*-Tg mice infected with SARS-CoV-2 at a high titer (5 × 10⁴ PFU/mouse) or low titer (1 × 10^3^ PFU/mouse). The results showed that *Tslp* expression was elevated on day 7 in the high-titer infected group (Fig 5A). In the low-titer infected group, *Tslp* mRNA expression increased by approximately 17-fold by day 4 and remained elevated thereafter (Fig 5B). The expression of *Il4*, *Il13*, *Il25*, and *Il33* mRNA did not change on day 7 in the high-titer infected group (Fig 5A). In the low-titer infected group, *Il4*, *Il13*, and *Il33* mRNA expression increased within a few days after infection and then decreased; however, their expression increased again approximately 3 weeks after infection (Fig 5B). These findings suggest that SARS-CoV-2 infection may be associated with type 2 cytokine and epithelial alarmin responses in the small intestinal mucosa.

### Eosinophil-associated histological and molecular features

**Fig 6:**
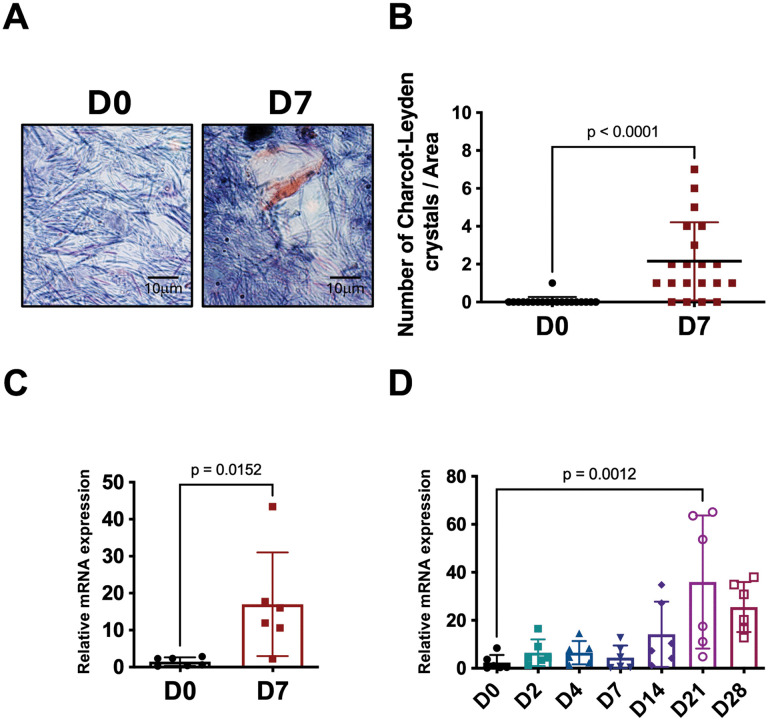
Formation of Charcot-Leyden crystals in the small intestinal mucosa after SARS-CoV-2 infection. (A) Papanicolaou staining of the intestinal contents during the absence of infection and 7 days after SARS-CoV-2 infection (5 × 10^4^ PFU/mouse) in the *K18-hACE2*-Tg mice. (B) Quantification of Charcot-Leyden crystals per area. n = 6 biological replicates. Quantitative reverse-transcriptase polymerase chain reaction (qRT-PCR) analysis of *Galectin10* in the small intestine in the uninfected state (D0), at 7 days (D7) after SARS-CoV-2 infection (5 × 10^4^ PFU/mouse) (C), and at 0, 2, 4, 7, 14, 21, and 28 days (D2, D4, D7, D14, D21, and D28) after SARS-CoV-2 infection (1 × 10^3^ PFU/mouse) (D) in the *K18-hACE2*-Tg mice. *Gapdh* was used as a loading control. n = 6 biological replicates.

To further examine eosinophil-associated intestinal responses during SARS-CoV-2 infection, we analyzed Charcot–Leyden crystal formation and Galectin-10 expression in *K18-hACE2*-Tg mice. Papanicolaou staining of small intestinal contents collected from SARS-CoV-2-infected mice revealed the presence of Charcot-Leyden crystals in the infected group on day 7 (Figs 6A and 6B). Real-time PCR analysis of *Galectin10* mRNA expression in small intestinal tissues showed increased expression in the high-titer infected group on day 7 (Fig 6C). In the low-titer infected group, *Galectin10* mRNA expression increased by approximately 15-fold at 2 weeks after infection and remained elevated thereafter (Fig 6D). Together, these results show the presence of eosinophil-associated histological and molecular features in the small intestine after SARS-CoV-2 infection.

## Discussion

In this study, we examined intestinal immune responses following SARS-CoV-2 infection in *K18-hACE2* transgenic mice using comprehensive gene expression analyses, histological evaluations, and real-time PCR. We found that SARS-CoV-2 infection was associated with a type 2-skewed immune profile in the small intestinal mucosa, characterized by increased *Il5* expression, eosinophil accumulation, and eosinophil-associated histological features, including Charcot-Leyden crystal formation. These findings suggest that SARS-CoV-2 infection may be accompanied by eosinophil-associated intestinal immune responses, which partially overlap with mechanisms observed in allergic and eosinophilic gastrointestinal disorders [17–20].

A key observation of this study was the marked upregulation of Il5 expression in the small intestine at day 7 post-infection. IL-5 is a central cytokine that regulates eosinophil differentiation, survival, and activation [21], and its elevated expression has been implicated in the pathophysiology of type 2 inflammatory diseases, including asthma and eosinophilic gastrointestinal disorders [18,19,22]. In parallel, increased expression of eosinophil-recruiting chemokines, including Ccl24 and Ccl11, was observed, supporting a role for coordinated cytokine–chemokine signaling in eosinophil accumulation within the intestinal mucosa [18,19,23]. Notably, high-titer infection was associated with earlier induction of Il5 and Ccl24, whereas low-titer infection showed delayed upregulation, suggesting that the magnitude of viral exposure may influence the kinetics of intestinal immune responses [9,15].

In the low-titer infection group, SARS-CoV-2 spike protein was detected in lung tissue by approximately day 7 after infection, whereas detectable spike protein was not observed in intestinal tissue during the early stage of infection (S2 Fig). It should be noted that the signal detected in S2 Fig represents SARS-CoV-2 spike protein and does not demonstrate the presence of infectious viral particles. The delayed induction of eosinophil-associated mediators in the low-titer infection group therefore raises the possibility that intestinal immune responses may occur even in the absence of detectable viral antigen in intestinal tissue. In this context, communication between the lung and gut, often referred to as the gut–lung axis, may contribute to secondary immune modulation in the intestine [24–26]. Previous studies have demonstrated that pulmonary viral infection can alter intestinal immune homeostasis through systemic cytokine signaling and neuroimmune pathways, even in the absence of detectable virus in the gut [24,26]. Although the present findings are consistent with this concept, direct causal relationships remain to be established. In particular, because K18-hACE2 mice develop severe pulmonary disease after SARS-CoV-2 infection, pulmonary inflammation and systemic immune responses may also have contributed to the intestinal immune changes observed in this study [9,15].

Histological analyses further showed increased eosinophil accumulation in the lamina propria of the small intestinal mucosa following SARS-CoV-2 infection. This observation was consistent with elevated expression of *Il5* and eosinophil-associated chemokines and supports the interpretation that cytokine and chemokine changes were accompanied by eosinophil recruitment [21,23]. In addition to eosinophil accumulation, we observed increased expression of *Tslp*, an epithelial-derived alarmin known to initiate type 2 immune responses in the context of epithelial stress and viral infection [17]. Persistent elevation of *Tslp*, particularly in the low-titer infection group, may reflect sustained epithelial perturbation and contribute to prolonged eosinophil-associated intestinal immune responses.

The temporal expression patterns of *Il4*, *Il13*, and *Il33* observed in this study suggest that intestinal immune responses to SARS-CoV-2 infection may change over time. Early transient increases in these cytokines may reflect acute epithelial stress responses, whereas later re-elevation may be associated with epithelial regeneration and tissue remodeling processes [17,25,27–32]. *Il33*, in particular, functions as an alarmin released upon epithelial damage and has been implicated in type 2 immune activation and tissue repair via ILC2 and Th2 pathways [17,27–29]. Notably, similar temporal patterns were observed across multiple type 2-associated cytokines, rather than in a single isolated marker, supporting the possibility of a coordinated temporal immune response. However, these findings do not definitively demonstrate distinct biological events, and cohort-to-cohort variability cannot be completely excluded.

An additional notable finding was the detection of Charcot–Leyden crystals in intestinal contents and the increased expression of Galectin10, which has been associated with eosinophil activation and extracellular trap formation [33,34]. Galectin-10 is the major protein component of Charcot–Leyden crystals, and Charcot–Leyden crystal formation is recognized as a characteristic feature of eosinophilic inflammation [33,34]. In addition, Charcot–Leyden crystal formation has been closely associated with eosinophil extracellular trap cell death [34]. The presence of these markers suggests that SARS-CoV-2-associated intestinal eosinophilia may involve eosinophil-associated inflammatory responses in the small intestinal mucosa. However, eosinophils and Charcot–Leyden crystals alone are not sufficient to establish intestinal allergy, and these findings may also reflect part of a broader inflammatory response. Therefore, the pathological significance of these eosinophil-associated changes should be interpreted cautiously.

Recent epidemiological studies have reported an increased risk of gastrointestinal disorders during the post-acute and long COVID phases, including motility disturbances, acid-related disorders, and functional GI conditions [5,35,36]. Persistent immune dysregulation, epithelial barrier disruption, and microbiome alterations have been proposed as contributing factors to these long-term outcomes [26,37–39]. In this context, the sustained and biphasic type 2 immune responses observed in the present study may provide a possible biological framework for understanding prolonged intestinal immune alterations after SARS-CoV-2 infection.

This study has several limitations. First, *K18-hACE2* transgenic mice exhibit severe pulmonary disease, which may influence systemic immune responses and limit direct extrapolation to human intestinal pathology [9,12,15,25]. Second, although eosinophil-associated cytokine and chemokine responses were evaluated at the mRNA level, cytokine protein quantification and functional assays were not performed. Therefore, eosinophil activation could not be directly demonstrated in this study. Third, although the presence of Charcot-Leyden crystals and increased *Galectin10* expression were consistent with features associated with eosinophil activation and possible extracellular trap formation, these findings do not directly prove eosinophil degranulation or ETosis. Fourth, the relative contributions of direct intestinal viral effects versus indirect immune modulation through systemic or gut–lung axis mechanisms could not be fully distinguished. In addition, the time-course analysis was based on independent cohorts at each time point rather than longitudinal sampling from the same animals; therefore, cohort-to-cohort variability cannot be completely excluded when interpreting the biphasic expression patterns. Finally, the RT^2^ Profiler PCR Array was performed with a limited number of biological replicates and was therefore used as an exploratory screening analysis. Key eosinophil-associated molecules identified by the array were subsequently examined by qRT-PCR, histological analysis, and immunohistochemistry using independent cohorts. Future studies incorporating cytokine protein quantification, flow cytometric analysis of eosinophil activation markers, spatial viral localization, epithelial lineage-specific analyses, and longitudinal immune profiling will be required to clarify these mechanisms.

## Conclusion

In conclusion, SARS-CoV-2 infection was associated with eosinophil-associated immune responses in the small intestine of *K18-hACE2* transgenic mice (S3 Fig). Although direct causality and definitive eosinophil activation were not established, these findings suggest that intestinal type 2–skewed immune alterations may occur after SARS-CoV-2 infection. This study provides a basis for considering eosinophil-associated intestinal immune responses as a potential component of gastrointestinal involvement in COVID-19.

## Supporting information

S1 Fig Confirmation of the *Il5* qRT-PCR products via agarose gel electrophoresis. The agarose gel electrophoresis was performed on the *Il5* PCR reaction solution obtained in the RT2 Profiler PCR Array (Fig 1), stained with ethidium bromide, and photographed with UV transilluminator.
(TIF)

S1 Text Raw image. Original unadjusted and uncropped image of the agarose gel electrophoresis shown in S1 Fig. The image shows the *Il5* qRT-PCR product obtained from the RT2 Profiler PCR Array presented in Fig 1A. This file contains the original unadjusted and uncropped image underlying S1 Fig.
(TIFF)

S2 Fig Histological analysis and SARS-CoV-2 spike protein detection in lung and small intestinal tissues after low-titer SARS-CoV-2 infection. (A) Hematoxylin and eosin staining of lung and small intestinal tissues collected at 0, 2, 4, 7, 14, 21, and 28 days after SARS-CoV-2 infection (1 × 10^3^ PFU/mouse) in *K18-hACE2*-Tg mice. (B) Immunohistochemical detection of SARS-CoV-2 spike protein in lung and small intestinal tissues collected at the same time points after infection.
(TIF)

S3 Fig Graphical abstract illustrating eosinophil-associated intestinal inflammation following SARS-CoV-2 infection. Illustrating features associated with SARS-CoV-2 infection in the small intestine.
(TIF)

S1 File SARS-CoV-2 infection protocol in *K18-hACE2* mice. (TIF)

S2 File List of target genes “cytokines and chemokines” for qRT-PCR array. (TIF)

## Abbreviations

ACE2: angiotensin-converting enzyme 2
BMP4: bone morphogenetic protein 4
CCL11: C-C motif chemokine ligand 11
CCL24: C-C motif chemokine ligand 24
COVID-19: coronavirus disease 2019
DAB: 3,3′-diaminobenzidine
DMEM: Dulbecco’s Modified Eagle Medium
EPX: eosinophil peroxidase
ETosis: extracellular trap cell death
GAPDH: glyceraldehyde-3-phosphate dehydrogenase
GI: gastrointestinal
H&E: hematoxylin and eosin
hACE2: human angiotensin-converting enzyme 2
HRP: horseradish peroxidase
IHC: immunohistochemistry
IL: interleukin
MBP: major basic protein
PBS: phosphate-buffered saline
PCR: polymerase chain reaction
PFU: plaque-forming unit
qRT-PCR: quantitative reverse-transcriptase polymerase chain reaction
RNA: ribonucleic acid
RT^2^: reverse transcription quantitative polymerase chain reaction
SARS-CoV-2: severe acute respiratory syndrome coronavirus 2
SD: standard deviation
TAE: Tris-acetate-EDTA
Tg: transgenic
TSLP: thymic stromal lymphopoietin
